# Nanoparticle Tracking Analysis: An Effective Tool to Characterize Extracellular Vesicles

**DOI:** 10.3390/molecules29194672

**Published:** 2024-10-01

**Authors:** Gabrielle Kowkabany, Yuping Bao

**Affiliations:** Chemical and Biological Engineering, The University of Alabama, Tuscaloosa, AL 35487, USA; gakowkabany@crimson.ua.edu

**Keywords:** extracellular vesicles, exosomes, nanoparticle-tracking analysis, fluorescent labeling

## Abstract

Extracellular vesicles (EVs) are membrane-enclosed particles that have attracted much attention for their potential in disease diagnosis and therapy. However, the clinical translation is limited by the dosing consistency due to their heterogeneity. Among various characterization techniques, nanoparticle tracking analysis (NTA) offers distinct benefits for EV characterization. In this review, we will discuss the NTA technique with a focus on factors affecting the results; then, we will review the two modes of the NTA techniques along with suitable applications in specific areas of EV studies. EVs are typically characterized by their size, size distribution, concentration, protein markers, and RNA cargos. The light-scattering mode of NTA offers accurate size, size distribution, and concentration information in solution, which is useful for comparing EV isolation methods, storage conditions, and EV secretion conditions. In contrast, fluorescent mode of NTA allows differentiating EV subgroups based on specific markers. The success of fluorescence NTA heavily relies on fluorescent tags (e.g., types of dyes and labeling methods). When EVs are labeled with disease-specific markers, fluorescence NTA offers an effective tool for disease detection in biological fluids, such as saliva, blood, and serum. Finally, we will discuss the limitations and future directions of the NTA technique in EV characterization.

## 1. Introduction

Extracellular vesicles (EVs) are membrane-enclosed particles naturally secreted by various cells [1]. EVs play significant roles in regulating cell–cell communication [2,3], directing cell behaviors [4,5,6], and are able to cross biological barriers [7]. Recently, numerous studies have shown that EVs are effective tools for disease diagnosis, such as neurological disorders [8,9,10], cardiac diseases [11], cancer progression [12,13,14], and metastasis [15,16]. However, the clinical translation of EVs is hindered by the lack of standardization in EV quantification [17]. The challenges of EV quantification result from their intrinsic heterogeneity. First, EVs have large heterogeneity in size (e.g., exosomes of 30–130 nm, microvesicles up to 1000 nm, and micro-sized apoptotic body) and EV cargos (e.g., miRNA and protein biomarkers) [18]. Second, EVs can be secreted from diverse sources, production conditions can vary greatly, and the secretion process is highly dynamic [19]. Many isolation and purification methods are available, such as ultracentrifugation, microfluidic separation, immunoaffinity capture, filtration, etc. [20,21,22,23,24]. All of these factors consequently lead to large variations and inconsistency in EV dosing during preclinical and clinical studies (e.g., 10^9^–10^14^ EVs/dose or 5 µg–6 mg proteins/dose). The inconsistency in dosing makes it impractical to cross-compare the therapeutic potentials of EVs. The large dose disparity further highlights the limitation in quantification methods during manufacturing and the crucial need to establish a consistent EV dosing strategy [25,26,27]. Therefore, there is an urgent need to establish consistency in EV quantification [28].

Many strategies have been developed to characterize EVs, and complementary techniques are typically used to obtain desirable information, such as transmission electron microscopy (TEM) for size and morphology, especially cryo-electron microscopy [29], enzyme-linked immunosorbent assay (ELISA), or flow cytometry and immunostaining for surface marker confirmation, and protein profiling by mass spectrometry [30,31]. Furthermore, emerging techniques continue to be developed, such as Raman tweezers microspectroscopy for chemical composition without labeling [29]. Regardless of potential applications, proper choices of characterization techniques for EV analysis are critical [32]. Among various characterization techniques, nanoparticle-tracking analysis (NTA) has proven to be one of the most useful methods for EV characterization, as it provides information about EV size, size distribution, and concentration in solution. In conjunction with fluorescent labeling of EV-specific biomarkers or disease markers, NTA in fluorescent mode allows for distinguishing different subpopulations and for disease detection in biological fluids.

In this review, we will discuss the NTA technique in both light scattering and fluorescent mode. In particular, we will highlight the factors affecting the specific information for NTA analysis. For example, practical considerations in the size and concentration measurement of EVs for light-scattering mode will be covered along with their practical applications. Beyond the size, size distribution, and EV concentration, it is critical to characterize protein and RNA markers to confirm EV phenotypes. With specific fluorescent labeling of EV protein markers, the fluorescent mode of NTA allows for the characterization of EVs in much specificity. The fluoresce-based NTA greatly depends on the fluorescent tags for EV labeling. The types of fluorescent tags not only influence the quality of NTA results but also define suitable applications. Here, special attention will be given to EV detection in biological fluids for potential disease diagnosis. Finally, we will discuss NTA’s limitations and future direction to address the need for standardization.

## 2. NTA Technique

**Figure 1:**
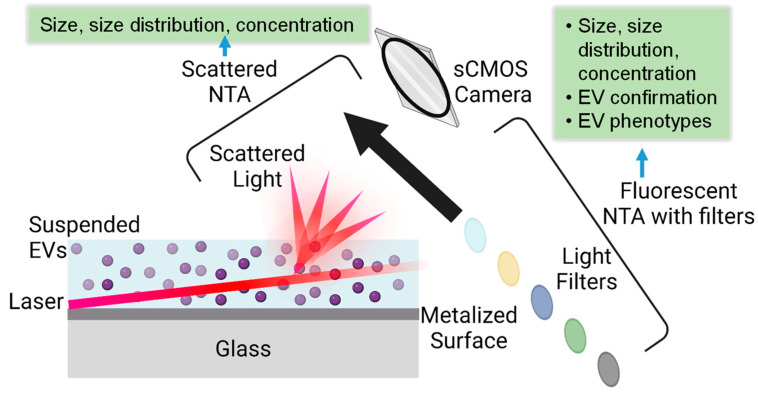
Illustration of NTA measurement principle in scattering and fluorescent modes along with obtained EV information, where the various colored circles indicated that different wavelength of emission lights can be collected by the selection of appropriate filter.

NTA is a widely used technique to visualize and characterize EVs, providing various information from a single technique, such as size, size distribution, concentration, surface biomarkers, etc. [33]. NTA tracks the Brownian motion of nanoparticles in a solution using a laser beam, which uses a high-sensitivity camera to capture the scattered light from nanoparticles in real-time. By analyzing trajectories of nanoparticles frame by frame within a certain period, the diffusion coefficient of each particle is calculated from its motion, and the hydrodynamic diameter is subsequently calculated using the Stokes–Einstein equation. Currently, there are two modes of NTA based on detecting signals either from the scattered lights from EVs or fluorescent emission of fluorescence-labeled EVs, as illustrated in Figure 1. NTA instruments currently have four choices of lasers (e.g., 405, 488, 532, or 642 nm).

Along with the appropriate filters, a wide range of fluorescent tags can be used to facilitate fluorescence measurements of EVs. The light-scattering mode NTA has the same working principle as dynamic light scattering (DLS), both of which track the Brownian motion of the objects by detecting the scattered lights [34]. DLS is another commonly used technique to characterize EVs in solution [32]. DLS only provides information on EV size and size distribution by analyzing the scattered light intensities. However, NTA generates a number-based size and size distribution by visualization. Compared to NTA, DLS does not offer information related to concentration or numbers because it analyzes all particles in solution simultaneously [35]. In addition, DLS has challenges in analyzing polydisperse samples, where scattered lights from larger objects dominant over the smaller ones. One benefit of DLS over NTA is the upper detection threshold of around 6 µm versus 1 µm for NTA [36]. In general, the size obtained from DLS is slightly larger than that from NTA because of the intrinsic heterogeneity of EVs and the domination of scattered light by larger-sized objects [37].

Typically, the light-scattering mode of NTA provides accurate size and concentration information in solution. The measured EV sizes typically agree well with other techniques, such as TEM [38]. However, sample conditions greatly affect NTA measurements, such as sample dilution, EV purity, measurement temperature, etc. In addition, the instrument settings, video acquisition, and data analysis settings all require calibration and optimization for consistent results [39]. It has been shown that the types of standards to calibrate the instrument were also important for EV measurement [39]. A comparison of measured EV size and concentration by the same operator using two NanoSight NS500 instruments at different laboratories suggested a large variation between EV size and concentration depending on applied instrument settings and technical conditions [40]. In the study, both intra-assay (within a day, n = 6) and inter-assay (day-to-day, n = 6) variations of EVs from different isolated methods were studied. It was suggested that the intra-assay variation, day-to-day variation, and operator variation were not significant on the same instrument. However, significant variation was observed between two instruments using identical software settings, which was attributed to instrument-optimized settings. This study emphasized the importance of consistent instrumental settings, standard operating procedures, and data analysis protocols. Therefore, proper sample preparation with standardized protocols is critical for NTA analysis of EVs to ensure accurate size and concentration. These conditions (e.g., sample dilution, choice of buffers, and removal of aggregates or contaminants) can significantly influence the NTA results. In addition, regular calibration of NTA instruments is essential to ensure accurate size and concentration measurements, such as camera settings, laser alignment, and temperature control. Finally, data acquisition and analysis parameters, such as frame rate, camera shutter speed, and tracking setting, also need attention. It is desirable that these parameters are optimized according to the sample to be measured to improve the reliability of the measurements. Therefore, it is critical to include instrumental settings and operating conditions when reporting experimental data of EVs from NTA.

When operated properly, NTA measurement provides fast and accurate information on EV size, size distribution, and concentration. For example, NTA-measured sizes of EVs isolated from conditioned media of human placenta-derived mesenchymal cells by differential centrifugation agreed well with the sizes obtained from TEM [38]. The size distribution obtained from NTA measurement provides insights into the heterogeneity of the EV populations because different types of EVs (exosomes, microvesicles, and apoptotic bodies) have distinct size ranges. However, the light-scattering mode of NTA cannot differentiate EVs from other nanosized objects, such as protein aggregates, contaminations, and salt precipitates. Consequently, the fluorescent mode of NTA was developed, which detects fluorescently labeled EVs. Switching from scattering to fluorescence mode, a long-pass filter is normally used to block the excitation wavelength. The fluorescent mode of NTA not only provides more accurate size and concentration information but also allows for the confirmation of EV types. When EV samples are analyzed in both light scattering and fluorescent modes, the percentage of EVs with labeled specific protein markers can be obtained in the solution. With the capability of multiple labeling, biomarker-positive or negative EVs allow the establishment of EV profiling as a supporting method for EV quantification.

In summary, NTA provides real-time EV analysis in solution, yielding accurate information regarding EV size, size distribution, and phenotypes. If EV samples are properly prepared and NTA instrument parameters are well calibrated, NTA quantification is highly valuable for a number of EV studies, such as the evaluation of isolation methods, dynamic studies of EV production and release, and EV detection based on surface markers. The selection of light-scattering mode vs. fluorescent mode will depend on the study needs and the information to be obtained.

## 3. EV Characterization with Light Scattering Mode of NTA

In the scattering mode of NTA, the solution sample is illuminated by a laser and the scattered light from the particles in solution is captured by a camera. The software algorithms analyze the particle trajectories to determine their hydrodynamic diameter and concentration. However, the light-scattering mode of NTA does not provide EV-specific information. Compared to other techniques, such as TEM and immunostaining, NTA is noninvasive with quick data acquisition, offering an effective tool for quickly comparing EV isolation methods, storage and EV release conditions. Numerous EV studies have utilized NTA as the primary characterization tool for EV size and concentration measurement. First, the scattering mode of NTA provides a high-resolution size distribution profile, enabling the characterization of the heterogeneity of EV populations based on the size distribution (e.g., exosomes, microvesicles, and apoptotic bodies). For example, NTA has a typical measurement size range of 50 nm~1 µm [36] with improved upper bound size around 2 µm for carefully prepared samples [41]. Compared to exosomes and microvesicles, apoptotic bodies are much larger (up to 5 µm) [42,43], which directly affects the measurement accuracy because of the lower particle number per analyzing volume and possible particle overlaps [36,37].

On the other hand, exosomes smaller than 50 nm are challenging for NTA to analyze. For uniform EVs less than 50 nm, DLS can provide a more reliable analysis of size and size distribution. Comfort et al. reported a detailed NTA protocol for quantifying the size and concentration of EVs isolated from mouse perigonadal adipose tissue and human plasma [33]. By analyzing EVs of different subpopulations from the human placenta and plasma using NTA, Soo et al. recommended several considerations for sample preparation [44]. First, the light-scattering mode of NTA has a low size detection limit of ~50 nm, smaller than which may not be quantified accurately. Second, NTA has an effective EV concentration of 10^8^–10^9^, but the accuracy of size measurement decreases for polydisperse samples. Tian et al. [45] recently presented a study to search critical parameters for the standardization of size and concentration determination of nanomaterials by NTA using polystyrene and SiO_2_ standard nanoparticles. The search result suggested an optimal concentration of 10^8^–10^9^ particles/mL. Therefore, a proper sample concentration is critical for NTA camera detection with all EVs that are detectable but without overlaying.

Because NTA provides accurate size and concentration information, the NTA technique has been the most useful tool to quantify EV yields from different conditions and monitor EV storage conditions. For example, the size and concentration of EVs isolated from human induced pluripotent stem cells and mesenchymal stem cells were accurately quantified by NTA [46]. NTA is also highly valuable for evaluating isolation efficiency and cross-comparison of EV isolation methods of various samples [47]. The NTA size measurement of EVs isolated from conditioned media of placenta-derived mesenchymal cells had a good agreement with TEM results of negative stained EVs [36]. Another study by Ekström et al. [48] isolated EVs from lymphatic drain fluids of seven patients with breast cancer the day after axillary lymph node dissection and subsequently quantified EV concentration by NTA, along with other techniques. NTA EV concentration coupled with protein quantification was used to evaluate the efficiency of the EV isolation process, and the ratio of EV number and protein amount served as an indicator of EV purity. NTA concentration quantification of urinary EVs isolated from the urine of 32 patients with varying levels of albuminuria showed the need for cautious interpretation of NTA concentration of EVs in biological fluids containing high proteins [49].

The accurate size and concentration measurement of NTA offers an effective tool for studying the stability of EVs. EV membrane disruption will not only change EV size but also decrease EV concentration. For example, the storage conditions (e.g., −80, −20, and 4 °C) of EVs isolated from HEK293T cells were studied by NTA in scattering mode as a function of time [50]. With increasing storage time, the disruption of EV membranes led to a reduction of EV concentrations. Therefore, NTA-based concentration quantification was more accurate and effective in monitoring the integrity of EVs compared to bulk RNA or protein quantification. Accurate determination of EV concentration is also critical for dose-response studies in therapeutic applications. EV number per unit volume has been one of the most commonly used EV dosing strategies in preclinical and clinical studies, where NTA has been the primary technique used to quantify the dose of EVs [25].

Given that NTA provides EV size and concentration information in real-time in solution, it allows for monitoring of EV release, aiding in optimizing culture conditions and treatment times to enhance EV yield. Monitoring the dynamic production of EVs from cultured cells over time can provide insights into cellular processes and the effects of treatments. For example, the exosome and microvesicle release from human lymphoblastoid T-cell lines Jurkat and CEM were stimulated with known potentiators (ionophores monensin and A23187) and quantified by NTA [44]. NTA quantification suggested that the release of microvesicles and exosomes was dependent on the time and concentration of potentiators. In the same study, NTA was applied to effectively monitor microvesicle release from peripheral blood monocyte-derived dendritic cells treated with bacterial lipopolysaccharide.

Furthermore, NTA has served as a great tool to study EVs isolated from body fluids, such as saliva, whole blood, urine, etc. For example, NTA was shown to effectively detect neuronal EVs in saliva for early detection of neurodegenerative diseases [51]. Despite the demonstrated effectiveness of NTA in studying EV concentration in biofluids, the quality of the results is highly dependent on sample preparation and instrumentation settings. Because NTA in light-scattering mode cannot differentiate EVs from other nanoparticles, the quantification of EV size and concentration in biological fluids with high protein contents will further increase the difficulty. A study using NTA to analyze EVs from neat biofluids (human serum and pericardial fluid) of patients undergoing cardiac surgery and from healthy controls suggested that several factors affected the NTA results, such as contamination from lipoproteins, sample freeze–thaw cycles without filtration, video frame, and EV numbers per frame [52]. To ensure cross-comparison among various studies, it is important to establish standard operating procedures for sample preparation, purity, and reference methods and materials. Sometimes, the types of NTA instruments also contribute to the consistency variation among EV characterization. For example, Bachurski et al. have compared the accuracy and repeatability of EV analysis from two NTA instruments (Malvern’s NanoSight NS300 and Particle Metrix’s ZetaView) [53]. In this study, the size and concentration of EVs from human serum and cell culture supernatants were compared as a function of serial dilutions for sample preparation and freeze–thaw cycles. The results suggested that ZetaView provided a more accurate and repeatable depiction of EV concentration, whereas NanoSight NS300 supplied size measurements of higher resolution.

NTA has been demonstrated to be an effective tool to quantify the size and concentration of EVs in cell culture and human body fluids, which has been explored as a diagnostic tool for disease detection. A study by Roni et al. compared EV concentration in patients’ saliva based on NTA quantification, which could be directly correlated to the progression of cognitive impairment in Alzheimer’s disease [54]. This study suggested that NTA-based EV quantification could be potentially used for early detection and cost-effective screening of diseases. Similarly, it was also shown that NTA could be used to detect EVs in other biological fluids, such as urine [55]. However, NTA is typically not very effective in analyzing small-sized EVs. Parsons et al. [56] developed a protocol to improve the precision and reproducibility of NTA to study EVs in the range of 50–120 nm in plasma [56]. It was suggested that increasing video replicates led to a reduction in overall variance and increased reproducibility.

In summary, the light-scattering mode of NTA is a non-destructive technique, allowing for further analysis of the same sample. The fast and accurate size and concentration measurement of NTA has demonstrated effectiveness in comparing EV isolation methods, studying EV purity and storage conditions, and monitoring EV production and release. In addition, NTA measurement highly depends on the EV isolation methods and sources. To ensure consistent results, many factors need to be considered, such as standard sample preparation protocols, consistent instruments, data acquisition and analysis settings. Despite the indisputable benefits in characterizing EV size, size distribution, and concentration, NTA in the light-scattering mode cannot differentiate the EVs from other nanoobjects, such as protein aggregates and other contaminants, which indicates the need for fluorescent NTA.

## 4. EV Characterization with Fluorescent Mode of NTA

The fluorescent mode of NTA is an advanced version of the NTA technique, which combines both scattered light and fluorescence detection. This approach is particularly powerful for confirming EVs through labeling EV-specific protein markers. In the fluorescent mode of NTA, EVs can be labeled with fluorescent molecules that specifically bind either to typical EV markers, such as tetraspanin (e.g., CD9, CD63, and CD81) or cytosolic protein (e.g., TSG101, ALIX, and syntenin) or target a specific disease protein marker. Switching from scattering to fluorescence mode, a long-pass filter is used to block the excitation wavelength and fluorescent emission from the labeled EVs are detected. Beyond the size and concentration, the fluorescent emission from the labeled EVs allows for the differentiation of labeled EVs from non-labeled ones in a mixed population. The most determent factor for fluorescent NTA is EV labeling (e.g., types of tags and labeling methods). In this section, we will first discuss EV labeling and then review the practical applications of fluorescent NTA in EV analysis.

### 4.1. EV Labeling

Fluorescent labeling of EVs refers to the incorporation of fluorescent molecules into EVs. The fluorescent labeling enables direct visualization of EVs with high specificity and sensitivity using the fluorescent mode of NTA. Numerous methods have been developed to label EVs, which will be discussed in detail in terms of choices of fluorescent tags, labeling strategies, and suitable applications. Depending on the labeling biomarkers, fluorescence NTA can be applied to study EVs for diverse bio-applications in vitro and in vivo [57].

**Figure 2:**
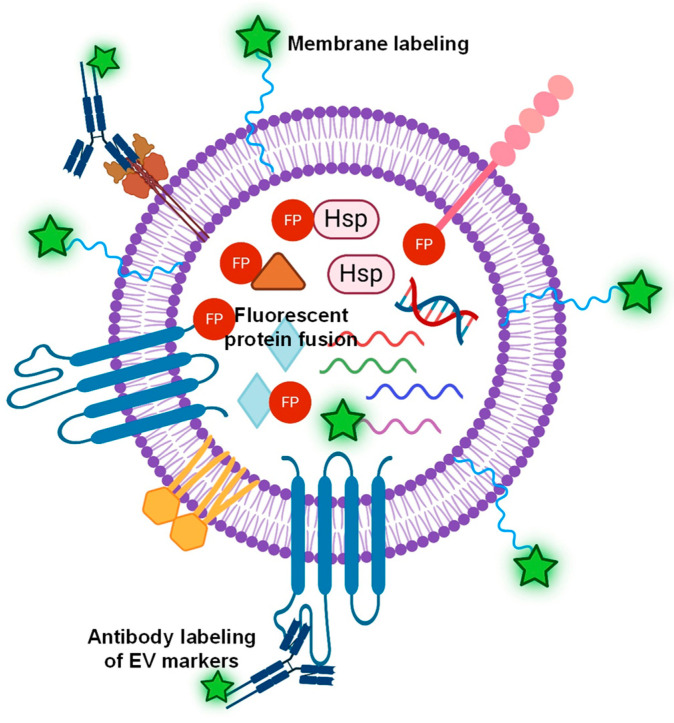
Illustration of various labeling strategies of EVs, such as lipophilic dyes for non-specific EV labeling, a fluorescent antibody targeting EV surface markers, or fluorescent protein (FP) fused to either surface proteins or internal cytosolic proteins, and mRNA labeling.

The fluorescent labeling of EVs can take place at the EV membrane, surface proteins, or internal cargos [58], as illustrated in Figure 2. First, the most common EV labeling is to use lipophilic dyes (e.g., PKH67, DiI, and DiO), which have a fluorescent head group and a long hydrophobic tail capable of inserting into EVs [59]. Membrane dye labeling has high labeling efficiency with strong fluorescent signals and minimal interference of internal cargo. In addition, the labeling process is relatively straightforward and quick [57] with developed labeling protocols [60]. Because membrane-labeling dyes stain all EVs, the quantification of EVs labeled with membrane dyes by fluorescence NTA provides the total counts of EVs regardless of phenotypes. On the other hand, the membrane dyes are non-specific and label any lipid-containing particles; therefore, the purity of the isolated EVs is critical to using membrane dyes as EV labels. For example, Chen et al. [61] systematically investigated the correlation of labeling efficiency with EV purity, suggesting that a 60–80% labeling efficiency for EVs isolated from conditioned cell culture media with EV purity of ~88% and ~40–70% labeling efficiency for EVs isolated from platelet-free plasma with EV purity of ~73%. In addition, the insertion of dyes into the lipid bilayer of EVs may affect their structure and function. The higher the labeling efficiency, the brighter the fluorescent signal, but a significant amount of lipophilic dye insertion could alter the size and structure of EVs. It was shown that excessive labeling with PKH26 could alter the membrane integrity of EVs [61]. Compared to PKH26, di-8-ANEPPS and DiI showed higher and more uniform EV labeling, with nearly 100% labeling efficiency for di-8-ANEPPS and 70–100% for DiI [61]. Unfortunately, dye aggregates and micelle structures were observed for all tested membrane dyes in this study [61], indicating a general issue of the membrane dyes for EV labeling.

Similarly, Midekessa et al. [62] investigated the effects of labeling conditions of lipophilic CellMask™ Green dyes on EVs isolated from human choriocarcinoma cells and different biological fluids using fluorescence NTA. By comparing EVs purified with different methods and EVs from biological fluids, it was found that the increased dye concentration significantly decreased the observed EV size. This observed size shift indicated the need to emphasize the optimization of dye concentrations to preserve the size of EVs after labeling. However, another comparison study [63] by fluorescent NTA showed that EVs labeled with lipophilic fluorescent dye molecules increased EV sizes at all labeling conditions. In contrast, a membrane-permeable dye that localized inside EVs did not affect the EV size, such as CFSE (5-(and-6)-carboxyfluorescein diacetate succinimidyl ester).

In addition to the alteration of EV structure, the labeling conditions must be carefully evaluated in salt-containing buffers or media, where most of these lipid dyes generally have low water solubility, readily forming micelles or aggregates. A study by Cha et al. [64] described a facile method with efficient EV labeling using lipophilic fluorescent dyes by adjusting the ionic strength of buffers with NaCl. Using cell-derived EVs labeled with Dil as a model system, it was found that a low-salt condition (20 mM) improved EV-labeling efficiency by a factor of 290 compared to high-salt concentration (150 mM). Another concern about using lipophilic dyes for EV labeling is the complete removal of unincorporated dyes that could cause background fluorescence [65]. A systematic study of EV purification efficiency after the labeling with various purification methods was performed using fluorescent mode NTA. It was found that the purification step must be carefully performed to ensure the accurate characterization of labeled EVs [66]. Therefore, the proper selection of purification methods and labeling conditions are important to membrane dye labeling. It was shown that increasing NaCl concentration in purification buffers after labeling induced aggregate formation of free dye molecules, which could be effectively removed through filtration [64]. Therefore, using a high salt concentration to reduce off-target labeling could be developed into a general method [64]. Besides the advancement of labeling and purification methods, new lipophilic fluorescent dyes have also been explored. Shimomura et al. [67] recently reported the development of a new type of dye consisting of a fluorescent cyanine group with an attached amphiphilic moiety that showed excellent EV labeling with no aggregation and less EV alteration based on the characterization by fluorescent NTA [67]. Alternatively, the fluorescent probes can be encapsulated into the lumen of EVs by incubating dye molecules permeable to bilayer membranes or nanoparticles by physical diffusion or active uptake, electroporation or sonication [68]. It was found that luminal labeling of EVs has less effect on the EV sizes [59].

One of the main drawbacks of membrane dye labeling is that it is non-specific, which means it cannot provide EV-specific information. Therefore, to obtain EV-specific information, the surface protein markers of EVs are commonly labeled with fluorescent antibodies specific for biomarkers of interest. By far, many different fluorescent antibodies have been developed against EV-specific surface markers (e.g., CD63, CD81, and CD9) that are characteristic of the EVs of interest. When EV-specific antibodies are conjugated to fluorescent dyes (e.g., FITC or Alexa Fluor—AF), EVs can be labeled directly and visualized by fluorescent NTA. For example, Cho reported the multifluorescence of single EVs and direct characterization by a modified form of fluorescent NTA [69]. Specifically, EVs were labeled with a blue fluoresce dye (AF405) conjugated antibody against CD81, a green fluorescent dye (AF488), a conjugated CD9 antibody, and a red fluorescent dye (AF647) CD63 antibody. The labeled EVs were subsequently characterized with fluorescent NTA with three excitation wavelengths (405, 488, and 638 nm). This study not only demonstrated the effectiveness of specific EV labeling via EV marker antibodies but also showed the feasibility of characterization of individual EVs using the fluorescent mode of NTA. The fluorescent antibody labeling provides high specificity, enabling specific detection and analysis of labeled EVs within a heterogeneous population. It also provides quantitative data on the concentration and size distribution of specific EV subpopulations. Therefore, this labeling strategy is best suited for EV confirmation and detection. For therapeutic applications or cell–cell communication studies, labeling surface protein markers may hinder the biological functions of EVs. In addition, the effectiveness of labeling with fluorescent antibodies depends on the antibody specificity, number of protein surface markers, and fluorescent stability.

Alternatively, EV-specific surface proteins or structural proteins within EVs can be tagged through genetic modification of EV-producing cells, yielding fluorescent protein-fused EV proteins. Fluorescent protein can be fused to EV protein markers either as recombinant proteins on the EV membrane or as internal cargo proteins in the EV lumen. Once established, fluorescent labeling is consistent and reliable. This labeling strategy enables facile functional analysis of EV components. For example, green fluorescent protein (GFP) has been fused to typical EV markers, CD63 [70] and CD81 [71], allowing for quantification and subpopulation determination of EVs by fluorescent mode NTA. However, this EV-labeling process requires genetic modification of the parent cells, which can be time-consuming and not applicable to all EVs. For example, the CD81 genes of humans (Hus) or Chinese hamsters (CHOs) were fused with GFP via a flexible peptide linker and then were expressed in CHO cells as recombinant proteins. Subsequently, GFP-labeled EVs were isolated from those transfected CHO cells, and EV concentration and yield across the EV purification process were quantified using the fluorescent mode of NTA. NTA concentration of EVs isolated from the CD81-GFP (CHO) and CD81-GFP (Hu) cell lines showed that the CD81-GFP (CHO) cell line did not secrete non-fused GFP while the expression of non-fused GFP from the CD81-GFP (Hu) cells was observed. EV concentrations were higher in the CD81-GFP (CHO) cells compared to the CD81-GFP (Hu) cells. For fused fluorescent protein fusion EV labeling, a systematic study of EVs with GFP fused CD63 showed the highest fluorescent protein per EV no more than 40–60% [72]. Some of the GFP-tagged EV showed quenched fluorescence either due to non-EV particles or the presence of truncated GFP. In addition, GFPs typically suffer from background interference, while RFPs are less bright. Most recently, a study by Mao et al. demonstrated effective EV labeling with a super-bright far-red fluorescence protein, mKate2 [73]. This dye is considered the next-generation fluorescent label with super brightness and far-red emission, high pH stability and photostability, low cytotoxicity, and low background interference. The fluorescent mode of NTA showed effective incorporation of mKate2-tagged protein into EV membranes with 90% labeling efficiency and without altering the EV size [73]. Additionally, the EV surface proteins can be labeled directly via click chemistry [74,75] to covalently attach fluorescent probes to the EV surface. This strategy can utilize a wide range of fluorescent probes with numerous linkers being developed [68]. However, chemical modification may affect the functionality and integrity of EVs.

Labeling EV protein markers is somehow limited by the number of surface proteins for enough fluorescent signals. Although fluorescent labeling with membrane dyes (e.g., PKH27, PKH67, etc.) provides an adequate signal for EV detection, this approach is non-specific and a higher amount of dye labeling was shown to alter the EV size and structural integrity. Recently, a study by Baldwin et al. [76] demonstrated the feasibility of determining the miRNA content of EVs isolated from A549 lung cancer cells using the fluorescent mode of NTA. In this study, miR-21 was fluorescently labeled by the hybridization of miRNA with fluorescently labeled, miRNA-specific molecular beacons encapsulated in cationic lipoplex nanoparticles that fuse non-specifically with negatively charged EVs. A stoichiometric analysis of miR-21 in EVs released from A549 lung cancer cells was analyzed in relation to the total EV population from the light-scattering mode of NTA. The result reported a threshold number of 33 miR-21 copies per EV required for fluorescence tracking. This study demonstrated the feasibility of fluorescent NTA analysis of fluorescent-labeled miRNA, which could potentially be used for EV sizing, concentration quantification, phenotyping, and identifying specific EV subpopulations in patient samples for diagnostic applications.

As discussed above, numerous fluorescent tags and labeling methods have been developed with their own pros and cons [77], as summarized in Table 1. By far, the photostability of most dyes remains a major concern for long-time tracking [78]. A study by Thane et al. [79] demonstrated reproducible NTA size and concentration quantification of fluorescently labeled EVs with quantum dots (QDs). In conjunction with the light-scattering mode, this study suggested that NTA offered an effective tool to estimate EV fraction with a specific biomarker. QDs are nanocrystals with bright and stable fluorescent emission, ideally suited for NTA applications. However, conjugation of larger inorganic nanoparticles could affect antibody activities.

In summary, choosing an appropriate fluorescent labeling method for EVs depends on the specific requirements of the study, such as the need for specificity, sensitivity, and functional integrity. It is important to balance these factors to achieve reliable and meaningful results.

### 4.2. Application of Fluorescent Mode of NTA in EV Characterization

The fluorescent mode of NTA is a powerful technique for EV characterization, offering insights into EV phenotypes, precise EV size profiles, etc. Therefore, fluorescent NTA has significant potential in the study of EVs for various in vitro and in vivo applications, such as accurate size and concentration quantification in a mixed solution and EV phenotype confirmation for disease detection. In this section, we will review the emerging applications of fluorescent NTA in EV characterization.

***Quantification of EV size and concentration***: Compared to the light-scattering mode, the fluorescent mode of NTA provides more accurate size, size distribution, and concentration of EVs, especially for EVs with low purity. For example, NTA number quantification of EVs from plasma via CD 63 antibody labeling showed a significantly higher concentration of EVs in plasma melanoma patients than in healthy donors [80]. This study suggested the potential of the fluorescent mode of NTA in disease detection. In another study, EVs isolated from the serum of hepatocellular carcinoma patients were labeled with lipophilic membrane dye and quantified by fluorescent mode of NTA for total EVs [81]. In the same study, isolated EVs were labeled with fluorescent antibodies against alpha-fetoprotein (AFP) and glypican (3GPC3) markers. NTA concentration quantification of GPC3- and AFP-positive EVs in hepatocellular carcinoma patient serum were compared to that from healthy controls, indicating a positive correlation of serum concentration of GPC3-positive EVs with tumor size. Therefore, this study demonstrated a proof-of-principle to use patient serum-derived EVs as a diagnostic tool. Furthermore, by comparing the fluorescently labeled EVs with the EV concentration of light-scattering mode, the fluorescent mode of NTA could effectively study contaminants and subpopulations [77].

***Confirmation of EV subpopulation***: In addition to accurate determination of size and concentration, NTA in fluorescent mode allows for the identification of EV phenotypes based on specific biomarker labeling [76]. EVs are intrinsically heterogeneous, where EV subgroups reflect various information related to biogenesis and characteristics of the secreting cells. Fluorescence-mode NTA was used to study purified and non-purified EVs from different cell sources, where the EVs were fluorescently labeled with both specific dyes and antibodies for EV identification. It was shown that the fluorescent mode of NTA effectively confirmed the presence of two EV subpopulations (85 nm and 135 nm) by tagging the typical EV marker, CD63, fused with GFP and expressed in Hela cell line [70]. In addition to the size and concentration of EVs, the fluorescent mode of NTA was shown to effectively detect EVs from human placental and plasma as small as ~50 nm and EV phenotypes were confirmed through labeling with QDs conjugated with NDOG2 antibody specifically against placental alkaline phosphatase [82].

EVs can be fluorescently labeled with non-specific membrane markers for total EV counts or labeled with antibodies specifically targeting surface markers of certain EV types, where the comparison of these two EV concentrations yields the accurate percentage of a specific EV type. For example, by proper labeling with fluorescent probes, fluorescent NTA provides a way to identify the size and concentration of an EV subpopulation in biological samples [83]. Another protocol development study [84] directly compared EV quantification by fluorescence NTA when EVs were labeled with either general membrane dye for total EV counts or with QD-conjugated antibody against disease-positive markers on EVs for quantification of certain EV groups [84].

**Figure 3:**
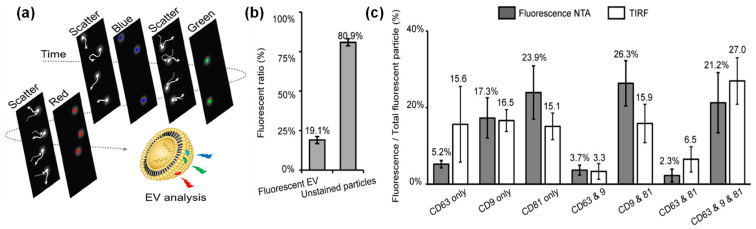
Single EV analysis by a multifluorescence NTA with time-sequential illumination where CD81, CD9, and CD63 markers were labeled by blue, green and red fluorescence, respectively: (**a**) illustration of the detection process, (**b**) relative percentage of EVs with and without fluorescent labels, and (**c**) comparison of detection of EVs with three CD markers by NTA and TIRF. (Copyright © 2021 American Chemical Society).

A study by Desgeorges et al. [85] reported the NTA quantification of EVs isolated from umbilical cord mesenchymal stromal cells using fluorescent mode. The comparison of EV concentrations from light-scattering mode versus fluorescence mode allowed for the determination of the percentage of protein marker-positive EVs within a mixed particulate solution. When EVs are labeled with multiple fluorescence tags, fluorescent NTA can detect multifluorescence from single EVs by a modified form of fluorescent NTA [69]. Using EVs derived from HEK293 cells as a model system, multifluorescence labeling was demonstrated by tagging CD81 with blue dye-conjugated antibody, CD9 with green fluorescent dye-conjugated antibody, and CD63 with red fluorescent dye antibody. EVs with multilabeling were analyzed effectively on a single EV level by a modified form of fluorescent NTA, where four lasers of different wavelengths are modulated in a time-sequential manner with on-off pattern (e.g., scattering with 520 nm laser → 405 nm laser excitation → scattering with 520 nm laser → 488 nm laser excitation → scattering with 520 nm laser → 638 nm laser excitation), as illustrated in Figure 3a. The scattering NTA mode provides total counts of EVs while florescent NTA mode generates EV counts of a specific group, such as 405 nm excitation for CD81-positive EV number, 488 nm excitation for CD 9-positive EV counts, and 638 excitation for CD63 EV counts. By analyzing the fluorescence detected by NTA, it suggested that only 20% of EVs had one or more EV-associated tetraspanin markers (CD9, CD69, and CD81), and the majority of EVs are not fluorescent (either EVs lacking those markers or contaminants) (Figure 3b). This NTA design enabled not only single EV characterization but also the determination of localization of EV-associated tetraspanin markers (CD9, CD69, and CD81) in a single EV.

The quantifications of EVs labeled with multiple CD markers were compared with results from total internal reflection fluorescence (TIRF) microscopy, resulting in results and trends for EVs labeled with single markers, dual markers, and triple markers (Figure 3c). TIRF microscopy is a highly effective optical tool that offers high resolution and instinctive results without additional processing [86]. Therefore, the comparison of these two techniques also inferred that the NTA is a reliable method for EV analysis with multiple labels.

***Monitoring EV release***: The accurate EV count allows fluorescent NTA to monitor the dynamic process of EV release and uptake in real-time, providing valuable information about their biological functions. For example, the fluorescent mode of NTA was applied to study the effects of cell culturing conditions on the release of large-sized EVs (e.g., microvesicles and apoptotic bodies) from human umbilical vein endothelial cells [87]. The NTA quantification suggested that cell density and conditioning time greatly affected the concentration and size distribution of EVs. In another study [88], the fluorescent mode of NTA was applied to study EV secretion from colorectal and glioblastoma cancer cells. shRNAs targeting RAB27A, a trafficking-associated protein, were known to facilitate EV secretion towards the plasma membrane. Therefore, shRNAs were used to alter EV release in colorectal and glioblastoma cancer cells. NTA quantification of EV concentration showed increased EV section under rotenone treatment or hypoxia conditions.

***EV detection in biological fluids***: The fluorescent mode of NTA is increasingly used in disease detection and diagnosis by studying fluorescently labeled EVs with specific disease markers. By leveraging the specificity and sensitivity of fluorescent labeling, disease-specific EVs provide valuable insights for early diagnosis. For example, EVs in urine carry specific markers of kidney damage, such as podocyte-specific proteins, e.g., podocin. By labeling both typical EV marker CD63 and disease-specific marker podocin with ant-CD63 and anti-podocin antibodies, fluorescent NTA effectively detected and quantified podocin-positive EVs in urine [55]. A study by Dlugolecka et al. [89] presented a comprehensive EV phenotyping using the fluorescent mode of NTA for EVs isolated from bronchopulmonary lavage fluid of patients with non-small cell lung cancer. The NTA quantification of EVs from bronchopulmonary lavage fluid was compared with patient plasma-derived EVs, where EVs were labeled with lipophilic membrane dye CMDR and fluorescent antibodies against CD63 and CD9. The NTA-based phenotyping of EVs in plasma and bronchoalveolar lavage fluid highlighted the importance of sample composition and EV purity to EV analysis by fluorescent NTA.

Furthermore, it was demonstrated that fluorescent NTA can be used to characterize EVs from human blood at a single particle level [90]. In this study, EVs were isolated from human whole blood and labeled with PKH67, a common lipophilic green fluorescent dye for membrane labeling. The NTA results were further validated by several typical EV surface markers, such as CD63, CD9, vimentin, and lysosomal-associated membrane protein 1. Similarly, small-sized EVs in saliva were effectively quantified by fluorescent NTA. The NTA quantification of EV concentration showed a much higher EV concentration in Parkinson’s disease patients than in the healthy control, serving as a diagnostic tool for PD [91]. This study further suggested that fluorescent NTA could be used as a potential tool to monitor disease progression with clinically acceptable sensitivity. However, a detailed analysis of small-sized EVs isolated from urine by several complementary methods suggested that the NTA evaluation of EVs is best supported by other methods [92]. In addition, the MISEV2018 guideline recommended that at least one tetraspanin (e.g., CD9, CD63, and CD81) and one cytosolic protein (e.g., TSG101, ALIX, and syntenin) should be detected for the positive identification of EVs. Moreover, the presence of non-EV-specific membrane markers (e.g., GM130) is an indication of potential contamination.

In summary, fluorescent mode NTA is a powerful tool for EV characterization, offering enhanced specificity and sensitivity compared to light-scattering mode NTA. It is particularly valuable for identifying and quantifying specific EV subpopulations, studying EV-cell interactions, and investigating the functional properties of EVs. It is important to optimize the experimental conditions to minimize operational effects from dye selection, labeling methods, and the needs of specific applications. The choice of fluorescent markers and the sensitivity of the NTA instrument are also critical for accurate detection and analysis.

## 5. Conclusions and Perspective

In summary, it is indisputable that NTA is a valuable tool for EV characterization, providing insights into EV size, size distribution, concentration, and heterogeneity. Many factors are important for NTA results in characterizing EVs, such as proper sample preparation in terms of EV concentration, presence of contaminants, instrumentation calibration (e.g., types, size, and concentration of calibration standards), and data acquisition and analysis parameters. Therefore, it is always a good practice to include an instrumentation setting when presenting NTA data in a publication. For the two modes of NTA, the light-scattering mode of NTA only provides size and concentration information but cannot differentiate EVs from other nanoparticles, such as protein aggregates. In contrast, the fluorescent mode of NTA offers a more accurate determination of the size and concentration of EVs because it only measures the labeled EVs. In addition, the fluorescent mode of NTA allows for the confirmation of specific types of EVs based on EV protein markers. However, the fluorescent NTA is highly dependent on the fluorescent labeling of EVs, which is affected by the labeling of fluorescent probes and labeling methods.

Despite some limitations, NTA continues to play a crucial role in advancing our understanding of EV biology and its implications in health and disease, such as the identification of EVs as disease markers, monitoring EV release and cell–cell communication. In particular, the fluorescent mode of NTA is highly valuable for disease detection by using EVs in body fluids. While NTA provides valuable EV information, it is often used in conjunction with other techniques, such as TEM, immunostaining, and flow cytometry, for comprehensive EV characterization. Continuous advancements in NTA technology will further enhance the importance of NTA in EV characterization for reproducible and comparable data across studies. First, it is important to improve the standardization and reproducibility of this technique in characterizing EVs through further advancement. These improvements can include (1) developing standardized protocols for sample preparation, data acquisition and analysis to ensure reproducibility across different laboratories, (2) establishing reference materials and calibration standards to improve the accuracy of NTA measurements, and (3) developing automated NTA systems to reduce user-dependent variability and increase throughput for large-scale studies. In addition, the sensitivity and resolution of this technique may be further improved, such as increasing the sensitivity of NTA to detect smaller EVs (<30 nm) and applying computational methods to enhance the resolution and accuracy of size distribution profiles.

Furthermore, integrating NTA with other techniques may offer additional possibilities, such as Raman spectroscopy, surface plasmon resonance, and mass spectrometry, to provide comprehensive characterization of EVs, including their molecular composition and surface markers. Finally, exploring the possibilities of integrating NTA with artificial intelligence by implementing machine-learning algorithms to enhance data analysis and automating particle tracking will provide deeper insights into EV populations and their heterogeneity.

## Figures and Tables

**Table 1:** A summary of various EV-labeling strategies.

Dye Types	Binding Location	Advantages	Disadvantages	Example
Lipophilic dye	EV membrane	High labeling efficiency, easy operation	Non-specific, aggregation, alteration of EV size and structure	Refs. [52,53,54,55]
Membrane permeable probes	EV lumen	Easy operation, minimal alteration of EV membrane	Non-specific, labeling efficiency-dependent labeling methods, possible EV structural disruption	Refs. [52,56,61]
Fluorescent protein fusion	EV surface or cytosolic proteins	High specificity and high efficiency, minimal alteration of EV protein function	Not applicable to all EVs, genetic modification of cells, and non-fused proteins.	Refs. [62,63,64,65,66]
Fluorescent antibody labeling	EV surface protein markers	Highly specificity	Limited number of surface proteins, alteration of protein function	Refs. [62,69]
Dye conjugation	EV surface proteins	High efficiency, versatile	Protein function alteration, precise chemical modification	Refs. [67,68]
